# Comparative pharmacokinetics of Theracurmin, a highly bioavailable curcumin, in healthy adult subjects 

**DOI:** 10.5414/CP204058

**Published:** 2021-08-23

**Authors:** Hyewon Chung, Seo Hyun Yoon, Joo-Youn Cho, Hee Kyung Yeo, Dongseong Shin, Ji-Young Park

**Affiliations:** 1 Department of Clinical Pharmacology and Toxicology, Korea University Guro Hospital,; 2 Department of Clinical Pharmacology and Therapeutics, Seoul National University College of Medicine and Hospital,; 3 Department of Biomedical Sciences, Seoul National University College of Medicine,; 4 R&D Center, Handok Inc.,; 5 Department of Clinical Pharmacology and Toxicology, Korea University College of Medicine, Korea University Anam Hospital, Seoul, and; 6 Department of Pharmacology, Gachon University College of Medicine, Gil Medical Center, Incheon, Korea; *These authors contributed equally to this work as corresponding authors.

**Keywords:** curcumin, bioavailability, clinical trial

## Abstract

Objective: Theracurmin is a submicron dispersed formulation of curcumin, which was developed to increase the bioavailability of curcumin. This study aimed to compare the pharmacokinetics of curcumin administered as two Theracurmin powder products and unformulated curcumin powder. Materials and methods: This randomized, three-treatment, six-sequence, and three-period crossover study enrolled 24 healthy subjects. Blood sampling was done until 12 hours after the administration of Theracurmin and curcumin powder to assess pharmacokinetics using a non-compartmental method. The plasma concentration of curcumin was determined using high-performance liquid chromatography coupled with tandem mass spectrometry. Results: The median time to reach the maximum concentration was 1.5 – 3 hours for Theracurmin and 8 hours for curcumin powder. The two Theracurmin products showed systemic exposure profiles that were comparable to each other. The exposure ratio of Theracurmin to curcumin powder was 18.4 – 20.5 for the maximum plasma concentration and 35.9 – 42.6 for the area under the concentration-time curve from dosing to the last measurable time. Conclusion: In conclusion, this study showed similar systemic exposure between the two Theracurmin products. The absorption of curcumin after the administration of Theracurmin was significantly enhanced compared with curcumin powder.

Clinical trial registry: NCT04028739 (clinicaltrials.gov) 

**What is known about this subject**

- Curcumin is well known for its low bioavailability.
- Theracurmin, which is curcumin dispersed with colloidal submicron particles, is one of the bioavailable curcumin formulations.

**What this study adds**

- This study contributes to enhance our knowledge on pharmacokinetic profiles of Theracurcumin compared with that of unformulated curcumin in a randomized, crossover study.
- This study shows that two marketed products of Theracurmin exhibit a systemic exposure similar to each other and were significantly absorbed compared with the unformulated curcumin.

## Introduction 

Curcumin, an ingredient in turmeric and long used as a spice, has emerged as a therapeutic agent. Studies have reported its beneficial effects on neurological, cardiovascular, lung diseases, metabolic syndrome, and liver disease mainly through anti-inflammatory and antioxidant mechanisms [1]. Furthermore, its role as an anticancer agent by exerting immunomodulatory effects has been suggested in a wide range of tumors such as breast, hematological, gastric, colorectal, pancreatic, hepatic, and prostate cancers [2]. 

Despite the promising biological activity, its clinical application has been limited due to its low bioavailability. Curcumin is almost insoluble in water and susceptible to degradation, especially under alkaline conditions [3]. In addition, it undergoes extensive metabolism such as glucuronidation and sulfation for excretion [4]. Several formulations have been developed to overcome the low bioavailability using phospholipid complexes, microemulsions, liposomes, polymeric micelles, and nanoparticles [5]. 

Theracurmin, which is curcumin dispersed with colloidal submicron particles, is one of the bioavailable curcumin formulations [6]. The average particle size of Theracurmin was considerably smaller (0.19 µm) than that of the conventional curcumin formulation (22.75 µm) and stably dispersed [7]. 

Theracurmin was initially developed as a liquid formulation, and few studies have evaluated its pharmacokinetic characteristics in humans. One of them compared the pharmacokinetics of Theracurmin to curcumin powder at a dose of 30 mg by administering each agent to 7 subjects. The results showed a 27.3-fold higher area under the concentration-time curve (AUC) after Theracurmin administration compared to curcumin powder administration [7]. A dose-escalation study reported the pharmacokinetics of Theracurmin from 150 to 210 mg in 6 healthy subjects. It showed that the plasma curcumin levels increased in a dose-dependent manner [8]. Another study also reported dose-dependent increases in plasma curcumin levels in cancer patients [9]. 

The formulation of Theracurmin has been improved to be available as powder form to reduce oral intake volume. Two different products of Theracurmin powder, which contain 90 mg (CR-033P) or 30 mg (CR-031P) of curcumin per capsule, have been available on the market. Recent clinical studies showed that the two Theracurmin products exhibited various beneficial effects such as improving memory and attention, ameliorating Crohn’s disease symptoms, and attenuating the indirect markers of muscle damage in humans [10, 11, 12]. 

Previously, plasma levels of curcumin after administration of CR-031P were reported to exhibit higher absorption than other curcumin preparations [6]. However, clinical data comparing the bioavailability of powder formulation of Theracurmin with unformulated curcumin is limited, and it is also unclear if the two products, CR-033P and CR-031P, have similar systemic exposure. 

Therefore, the aim of this study was to compare the bioavailability of curcumin administered as two different products of Theracurmin as well as curcumin powder and to explore the pharmacokinetic properties of curcumin. 

## Materials and methods 

### Investigational products 

Two Theracurmin products, CR-033P (TOYO capsule, Shizuoka, Japan) and CR-031P (BIHOLON, Toyama, Japan), were used in this study. Both were made of turmeric oleoresin curcumin after the extraction, dissolution, mixing, grinding, and homogenization of turmeric raw material [7, 8]. A capsule containing CR-033P consisted of 36% curcumin, 14.6% gum ghatti, 0.7% citric acid, 8.7% dextrin, and 40% maltose. A capsule containing CR-031P consisted of 12% curcumin, 3.2% gum ghatti, 0.27% citric acid, 54.53% dextrin, and 30% maltose. Briefly, the main difference between the two products was in the curcumin content, where 1 capsule of CR-033P contained 90 mg of curcumin and CR-031P contained 30 mg of curcumin. 

As a comparator, a capsule of unformulated curcumin powder containing 90 mg of curcumin was used. The curcumin powder was obtained by the same solvent extraction of turmeric raw material as Theracurmin without further processing. 

### Subjects 

Informed consent was obtained from all subjects prior to the study procedures. Healthy subjects underwent screening, including medical history, vital signs, clinical laboratory tests, and physical examinations. The subjects who met the inclusion/exclusion criteria were randomized into one of six sequences. Participants aged 19 – 60 years with a body mass index (BMI) of 18.0 – 30.0 kg/m^2^ were included. Subjects were excluded if they had clinically significant disease or a history of surgery or allergy; treated with medications or consumed foods containing turmeric within 7 days before the study; or participated in clinical study within 90 days before the first administration of investigational product. Female subjects who were pregnant or lactating at screening or planning to be pregnant during the study were also excluded. 

### Study design 

A randomized, open-label, three-period, six-sequence, crossover study was conducted. According to the randomized sequence, subjects were administered 1 capsule of CR-033P, 3 capsules of CR-031P, and 1 capsule of curcumin powder after overnight fasting. Each treatment period was separated by a washout period of at least 7 days. At each treatment period, blood sampling for pharmacokinetic analysis was done pre-dosing and at 0.5, 1, 1.5, 2, 3, 4, 6, 8, and 12 hours post-dosing. Adverse events were collected during the entire study period. The study was conducted in accordance with the principles of the Declaration of Helsinki and was approved by the Institutional Review Board of Korea University Guro Hospital, Seoul, Korea (Clinical trials registry number: NCT04028739). 

### Determination of plasma curcumin concentration 

Blood samples were centrifuged at 1,977×g for 10 minutes at 4 °C to separate the plasma, which was then stored under –70 °C until analysis. The plasma concentrations of curcumin were determined using high-performance liquid chromatography coupled with tandem mass spectrometry. Curcumin-d6 (Toronto Research Chemicals Inc., Toronto, ON, Canada) was used as an internal standard. The method was validated in terms of selectivity, calibration curve, accuracy, precision, dilution, carryover, and system suitability [13]. 

Thawed plasma samples (100 µL) were mixed with 50 µL of 0.2 M phosphate buffer (pH 7.2) and 30 µL of β-glucuronidase. After 1 hour of incubation at 1,500 rpm at 37 °C, 50 µL of internal standard (100 ng/mL in 100% methanol) and 1 mL of ethyl acetate were added and centrifuged at 18,341×g for 10 minutes at 4 °C. The upper layer of the mixture was transferred to a new 1.5 mL polypropylene tube, evaporated under nitrogen gas at 40 °C for 20 minutes, and reconstituted with 100 µL of 60% acetonitrile. After centrifugation, 4 µL of the supernatant was injected onto the Phenomenex Gemini C18 column (Phenomenex Inc., Torrance, CA, USA). The calibration curve was linear within the range of 1 – 500 ng/mL, with an accuracy of 88.92 – 114.6% and a precision of less than 9.956%. 

### Pharmacokinetic assessment and statistical analysis 

Non-compartmental analysis was used for the pharmacokinetic assessment using WinNonlin version 8.1 (Certara, St. Louis, MO, USA). The maximum plasma concentration (C_max_) and time to reach C_max_ (t_max_) were determined directly from the individual time-concentration profiles, while the area under the concentration-time curve from dosing to the last measurable time (AUC_last_) was calculated using the linear-up log-down trapezoidal method. 

Descriptive statistics were used to summarize the demographic data and pharmacokinetic parameters. To compare the pharmacokinetic parameters between the treatments, a linear mixed-effects model with period, sequence, and treatments as fixed effects and sequence-nested subject as a random effect was used. The point estimate of the geometric mean ratio (GMR) and its 90% confidential interval was calculated by applying the exponential function to the difference of least squares means between the two formulations. The association between demographic data and pharmacokinetic parameters was explored by correlation analysis. Statistical analyses were performed using SAS version 9.4 (SAS Institute Inc., Cary, NC, USA). 

## Results 

### Subjects 

A total of 26 subjects underwent screening, and 24 of them were enrolled. All 24 subjects were administered the three investigational products and completed the study. All subjects were male, with mean age, height, weight, and BMI of 29.9 years, 173.3 cm, 69.1 kg, and 23.0 kg/m^2^, respectively. 

### Pharmacokinetics 

**Figure 1.**
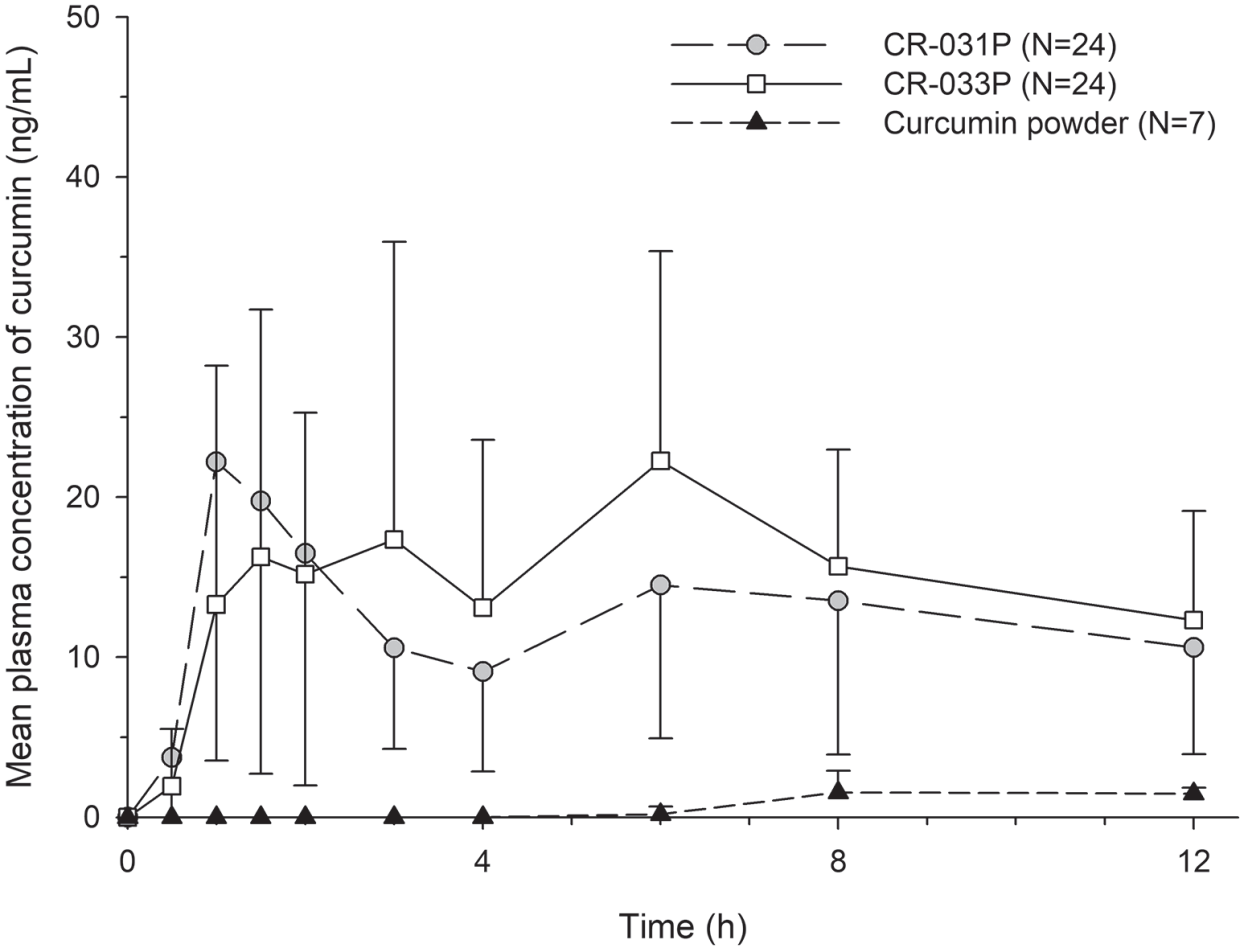
Mean concentration-time profile of plasma curcumin.

**Figure 2.**
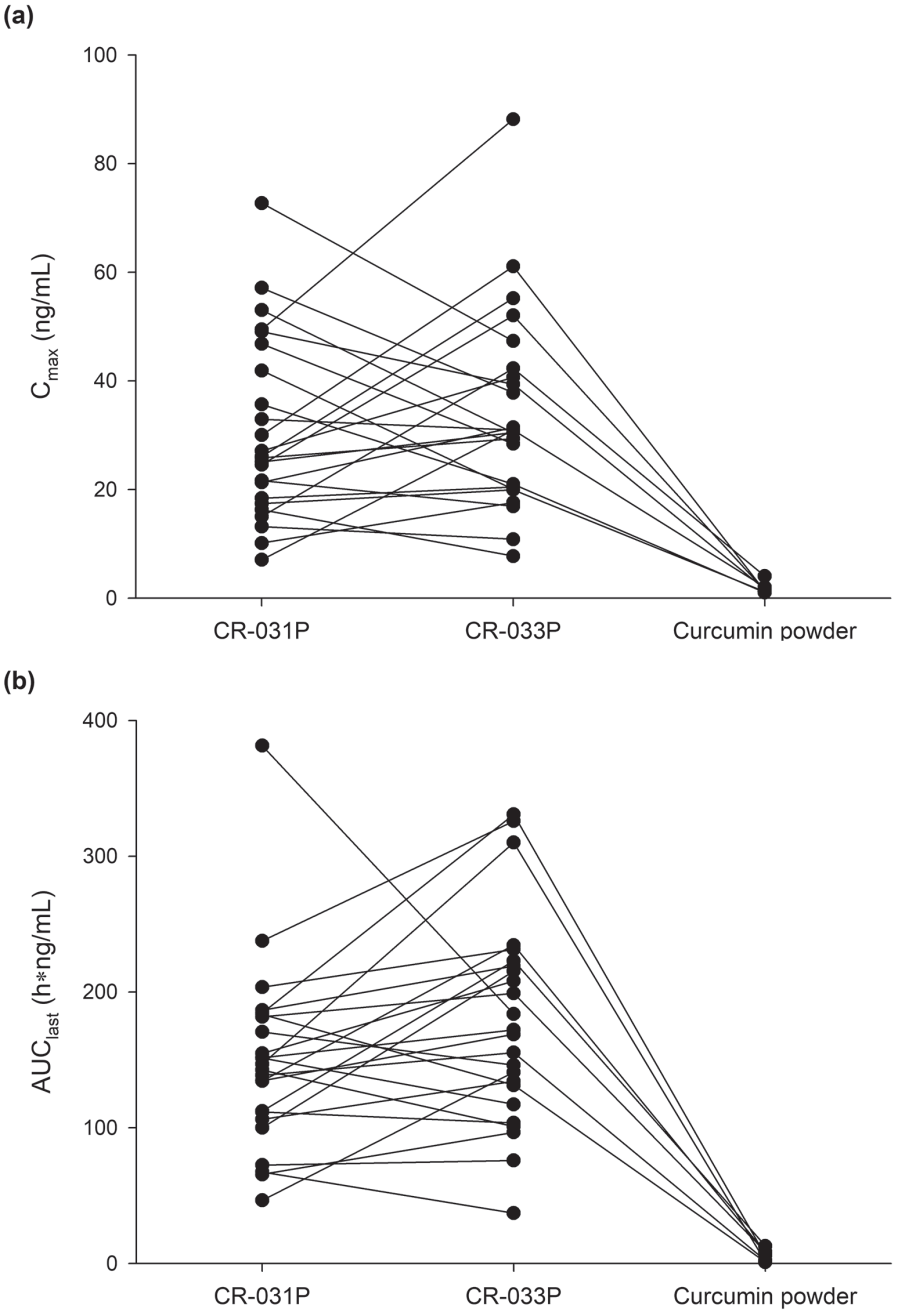
Comparison of systemic exposure of curcumin (a: maximum plasma concentration; and b: area under the concentration-time curve from dosing to the last measurable time).

CR-031P and CR-033P showed comparable time-concentration profiles, while the systemic exposure of curcumin powder was negligible (Figure 1). After taking CR-031P and CR-033P, all subjects had measurable concentrations reaching a median t_max_ at 1.5 hours and 3 hours, respectively. However, pharmacokinetic parameters were derived from only 7 subjects based on at least 1 measurable concentration of curcumin powder (Table 1). The comparison of the products including all 24 subjects with missing data revealed that the geometric least-squares mean ratios of CR-033P and CR-031P to curcumin powder for the C_max_ were 20.5 and 18.4, respectively, and 42.6 and 35.9 for the AUC_last_, respectively (Table 2) (Figure 2). Neither body weight nor BMI was significantly correlated with the C_max_ and AUC_last_ of curcumin (Supplemental Figure 1). 

### Safety 

No adverse events were reported during the study. Liver function tests such as aspartate transaminase, alanine transaminase, and γ-glutamyltransferase remained stable with mean values of 19.8, 19.2, and 32.8 IU/L at screening and 19.2, 20.1, and 33.0 IU/L, respectively, at the end of the study. There were no clinically significant findings in other laboratory tests, vital signs, or physical examinations. 

## Discussion 

Although curcumin is a widely used food ingredient and expected to have health benefits, the pharmacokinetic characteristics have been insufficiently identified. Several clinical studies reported pharmacokinetic parameters mostly to compare the systemic absorption of curcumin between newly developed bioavailable formulations and unformulated curcumin [14]. However, knowledge is limited due to sparse sampling, the small number of subjects, or the diversity of the study design such as sampling time points and analytical methods. In addition, the blood concentration of curcumin even after huge doses of curcumin was very low or undetected, which makes inferring pharmacokinetic characteristics from those observed after the administration of formulated curcumin necessary. 

The plasma concentrations of curcumin increased rapidly after Theracurmin administration with a median t_max_ of 1.5 – 3 hours. Concentrations increased earlier compared with curcumin powder, which resulted in no measurable concentrations until 6 hours post dosing. The results were consistent with other studies that reported the t_max_ of unformulated curcumin at 6 – 7.5 hours after administration [7, 15, 16, 17]. When the exposure was compared between Theracurmin and curcumin powder, the ratio was 18.4 to 20.5 for the C_max_ and 35.9 to 42.6 for the AUC_last_, respectively. In a previous study, the C_max_ and AUC were 16.0- and 27.3-fold higher after the administration of Theracurmin as liquid formulation compared with values after the administration of curcumin powder [7]. It should be noted that the previous report directly compared the mean values of the parameters obtained from independent subject groups, whereas our study adopted a crossover design to deal with inter-individual variability and used a linear mixed-effects model to include the period, sequence, and treatment as fixed effects. 

Interestingly, the time-concentration profile of plasma curcumin after Theracurmin administration showed a second peak at 6 hours post-dose. Considering that the subjects had lunch 4 hours after the administration, enterohepatic circulation may have produced the second peak. Previously, the second peak was also demonstrated in a study of rats after intravenous curcumin administration [18]. Another study reported that intravenous and intraperitoneal doses of curcumin were well excreted in the bile, supporting the enterohepatic circulation of curcumin [19]. 

Systemic exposure after curcumin administration showed significant variability with coefficients of variation from 43.05 to 77.66%. Body size such as weight or BMI is one of the factors that can explain inter-individual variability. BMI tends to affect the volume of distribution and consequently the exposure, especially for lipophilic compounds. However, there was no significant correlation between those and the pharmacokinetic parameters in our study. This might have resulted from limited distribution because curcumin exists mainly in its conjugated form after absorption [20, 21]. Since plasma level of free curcumin is expected to be undetectable, this study measured the total concentration of free curcumin and its glucuronide form by incubating plasma samples with β-glucuronidase. 

Although this study reported the systemic exposure of curcumin in humans, the results must be interpreted with caution. First, the pharmacokinetic parameters reported after curcumin powder administration may not reflect true pharmacokinetic characteristics, since most of the concentrations were not detected or lower than the lower limit of quantification. Seven subjects had at least 1 concentration data point, whereas 17 subjects had no measurable concentrations. Therefore, it is likely that the pharmacokinetic parameters of curcumin powder were biased to reflect only individuals who absorbed curcumin well. Furthermore, the 7 subjects had at most 3 measurable concentrations each, leading to insufficient pharmacokinetic analysis. When comparing the pharmacokinetic parameters, data from all 3 treatments including the missing values were used to fit the mixed-effects model. Therefore, missing values after the administration of curcumin powder may have yielded insufficient power for estimated GMR, especially in the comparison of CR-033P or CR-031P with curcumin powder. 

Second, although this study intended to enroll both male and female subjects aged 19 – 60, the subjects enrolled in our study were limited to healthy young male adults. One previous study reported a higher AUC of curcuminoids in women than in men, while another study reported no significant differences between genders [16, 22]. Although sex differences in the pharmacokinetics of curcumin are controversial, extrapolating the results to other subject groups such as females, elderly people, children, and specific patients needs a careful approach. 

## Conclusion 

In conclusion, this study explored the systemic exposure of curcumin after the administration of Theracurmin CR-033P, CR-031P, and unformulated curcumin powder in healthy male subjects. The two Theracurmin products showed a systemic exposure similar to each other and significantly enhanced absorption compared to the curcumin powder. 

## Authors’ contributions 

Research conception and design: HC and HKY; experiments: HC, SHY, and J-YC; statistical analysis of the data: HC and DS; interpretation of the data: HC and J-YP; writing of the manuscript: HC, DS, and J-YP; review of the manuscript: SHY, HKY, and J-YC. 

## Funding 

This study was sponsored by Handok, Inc. 

## Conflict of interest 

HKY is an employee of Handok, Inc. The other authors report no conflict of interest. 

**Table 1.** Pharmacokinetic parameters after administration of CR-033P, CR-031P, and curcumin powder.

	CR-033P (N = 24)			CR-031P (N = 24)			Curcumin powder (N = 7)		
	C_max _ (ng/mL)	AUC_last _ (h×ng/mL)	t_max _ (h)	C_max _ (ng/mL)	AUC_last _ (h×ng/mL)	t_max _ (h)	C_max _ (ng/mL)	AUC_last _ (h×ng/mL)	t_max _ (h)
Mean	33.78	177.55	3.92	30.75	148.62	3.08	1.80	5.75	9.43
SD	17.95	76.43	2.85	16.73	68.45	3.42	1.05	4.46	2.51
Geometric mean	29.50	160.01	2.99	26.49	135.00	1.99	1.62	4.00	9.14
Min	7.76	37.08	1.00	7.06	46.53	1.00	1.04	1.04	6.00
Median	30.73	170.50	3.00	25.95	144.74	1.50	1.43	5.91	8.00
Max	88.17	330.82	12.00	72.73	381.38	12.03	4.05	12.93	12.00

C_max_ = maximum plasma concentration; AUC_last_ = area under the concentration-time curve from dosing to the last measurable time; t_max_ = time to reach C_max_.

**Table 2.** Comparison of systemic exposure between CR-033P, CR-031P, and curcumin powder (N = 24).

Comparison	Geometric least squares mean ratio (90% confidence interval)	
	C_max_	AUC_last_
CR-033P to CR-031P	1.11 (0.90 – 1.38)	1.19 (0.96 – 1.47)
CR-033P to curcumin powder	20.5 (14.1 – 29.6)	42.6 (29.3 – 61.7)
CR-031P to curcumin powder	18.4 (12.7 – 26.6)	35.9 (24.7 – 52.1)

C_max_ = maximum plasma concentration; AUC_last_ = area under the concentration-time curve from dosing to the last measurable time.

## Supplemental material

Supplemental Figure 1.
