# Supplementary material for: Comparative pharmacokinetics of Theracurmin, a highly bioavailable curcumin, in healthy adult subjects
Source: Int J Clin Pharmacol Ther. 2021 Aug 23;59(10):684–90. doi: 10.5414/CP204058 (PMC9097512; doi:10.5414/CP204058)

Supplemental Figure 1. Correlation between demographics (a, b: body mass index (BMI); c, d: weight) and log transformed pharmacokinetic parameters (a, c: maximum plasma concentration (C_{max}); b, d: area under the concentration-time curve from dosing to the last measurable time (AUC_{last}))

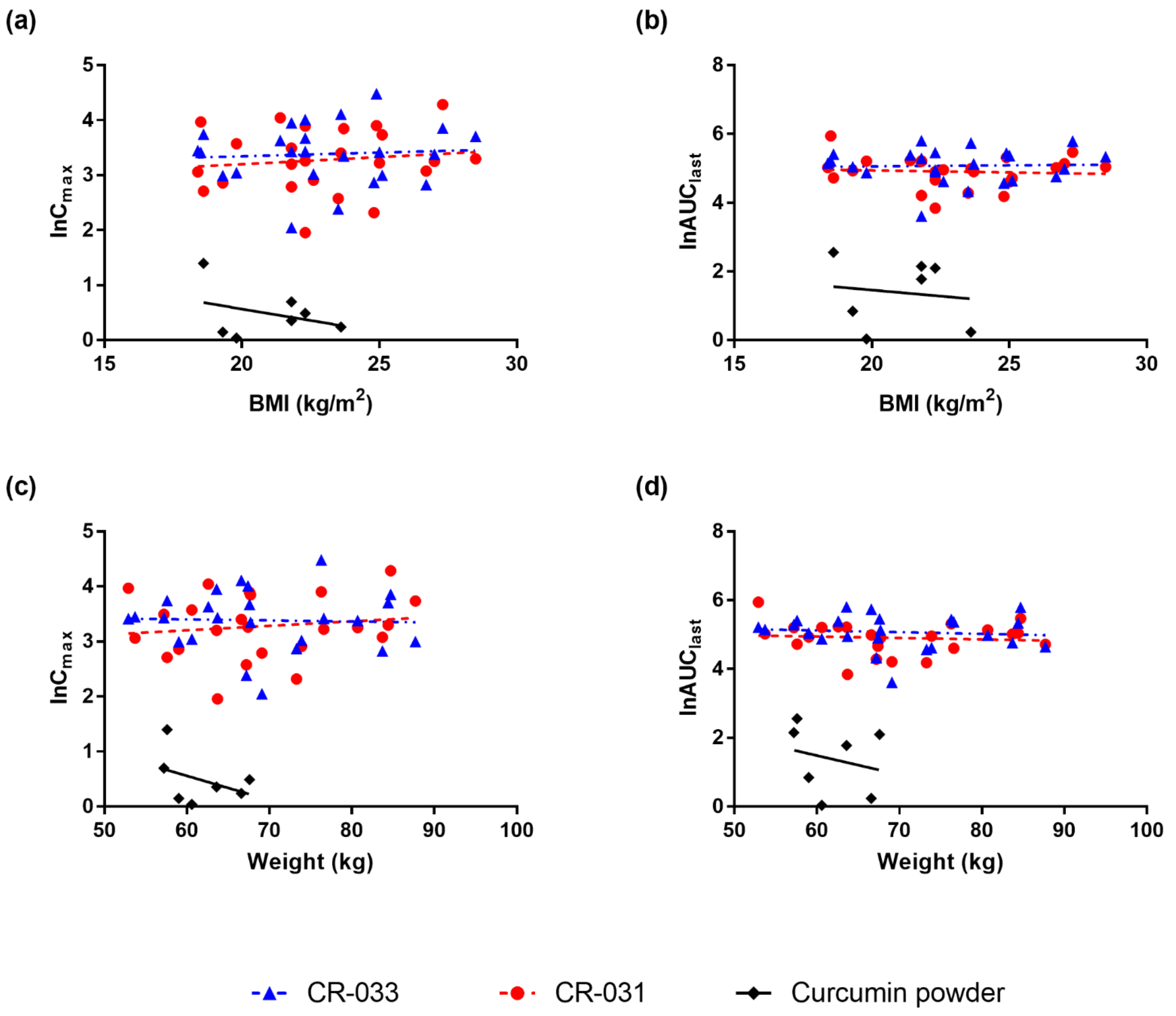

Supplement: Supplementary file 1 [file intjclinpharmacol-59-684-S01.pdf]
